# Health Status of the Eastern Grey Squirrel (*Sciurus carolinensis*) Population in Umbria: Results of the LIFE Project ‘U-SAVEREDS’

**DOI:** 10.3390/ani12202741

**Published:** 2022-10-12

**Authors:** Deborah Cruciani, Silvia Crotti, Daniele Paoloni, Valentina La Morgia, Andrea Felici, Paola Papa, Gian Mario Cosseddu, Livia Moscati, Paola Gobbi

**Affiliations:** 1 Istituto Zooprofilattico Sperimentale dell’Umbria e delle Marche “Togo Rosati” (IZSUM), Via G. Salvemini 1, 06126 Perugia, Italy; 2 Istituto Oikos srl, Via Crescenzago 1, 20134 Milano, Italy; 3 Istituto Superiore per la Protezione e la Ricerca Ambientale (ISPRA), Via Vitaliano Brancati 48, 00144 Roma, Italy; 4 Istituto Zooprofilattico Sperimentale del Lazio e della Toscana “M. Aleandri” (IZSLT), Via Appia Nuova 1411, 00178 Roma, Italy; 5 Istituto Zooprofilattico Sperimentale Abruzzo e Molise “G. Caporale” (IZSAM), Campo Boario, 64100 Teramo, Italy

**Keywords:** dermatophytes, invasive animal species, Italy, *Sciurus carolinensis*, zoonoses

## Abstract

**Simple Summary:**

Invasive alien species are non-native species introduced deliberately or unintentionally beyond their past or present natural distribution, and their introduction and spread threatens local biological diversity. The Eastern grey squirrel is native to North America and was introduced to the British Islands, Italy, and South Africa. Around the year 2000, a new population of grey squirrels was recorded in Perugia, central Italy, where the species populated an area of approximately 50 km^2^, both in woodland and urban areas. The Eastern grey squirrel represents a huge threat to the conservation of the native Eurasian red squirrel when the two species coexist. Moreover, given their confident behaviour with humans, the non-native squirrels can negatively impact public health. The U-SAVEREDS Project was set up for Eurasian red squirrel conservation in Umbria through the eradication of the alien species and it also provided information on the health status of the Eastern grey squirrel to identify any infectious agents. The recovery of zoonotic pathogens allowed to assess the Eastern grey squirrel’s impact on human and domestic and wild animals’ health, provide helpful feedback for the management and eradication procedures, and raise public awareness through environmental education.

**Abstract:**

The introduction of the Eastern grey squirrel (*Sciurus carolinensis*) in Europe is one of the best-known cases of invasive alien species (IAS) colonisation, that poses a severe risk to the conservation of biodiversity. In 2003, it was released in a private wildlife park near the city of Perugia (Italy), where it is replacing the native Eurasian red squirrel (*Sciurus vulgaris*). The LIFE13 BIO/IT/000204 Project (U-SAVEREDS) was set up for the *Sciurus vulgaris* conservation in Umbria through an eradication campaign of grey squirrels. One hundred and fifty-four animals were analysed for bacteriological, mycological, virological, and serological investigations (C4 action). Sanitary screening showed that *Sciurus carolinensis* is a dermatophyte carrier, and therefore, it could cause public health issues for humans, considering its confident behaviour. Moreover, it has been marginally responsible for the spreading of *Candida albicans*, *Coxiella burnetii*, and *Borrelia lusitaniae*. Health status evaluation conducted on the *Sciurus carolinensis* population indicated that it is necessary to raise awareness of its impacts on biodiversity and human health. Moreover, the health status and behaviours of the IAS must be considered when control or eradication campaigns are planned.

## 1. Introduction

Invasive alien species (IAS) are non-native species introduced deliberately or unintentionally beyond their past or present natural distribution, and their introduction and spread threatens local biological diversity [1]. The global rate of IAS introduction has greatly increased in the last few decades, which presents one of the most concerning threats to the conservation of biodiversity, second only to habitat loss and fragmentation [2,3]. IAS interact with native species through different ecological processes, including competition, predation, parasitism, disease vectoring, hybridisation, and spillover/spillback mechanisms [4,5]. Moreover, IAS can negatively impact public health and cause economic consequences [6]. Several zoonoses that have occurred in the last decade, such as SARS in Asia, MERS in the Middle East, Nipah in South-East Asia, Hendra in Australia, and SARS-CoV-2 worldwide, have highlighted the importance of monitoring animals as potential reservoirs or vectors of diseases. Recently, there has been increasing interest in the exploration of health risks, particularly posed by IAS, especially from scientists and policymakers [7,8,9,10]. To prevent the spread of IAS, it is necessary to collect as much information as possible regarding their dispersion [11].

One of the best-known cases of invasive species colonisation in Europe is the introduction of the Eastern grey squirrel (*Sciurus carolinensis*) [12,13,14]. This rodent was introduced to Italy and England from the USA, to South Africa and Ireland from England, and to Scotland from Canada. It was also introduced to Australia in the 1880s but is no longer present there [15]. In Umbria (central Italy), grey squirrels were unintentionally released in 2003, in a private wildlife park within the Special Conservation Area IT5210021 ‘Monte Malbe’, near the city of Perugia [16]. Its spread area reached approximately 50 km^2^ and is characterised by mixed deciduous forests and urban centres. This alien species is replacing the native Eurasian red squirrel (*Sciurus vulgaris*) in the British Islands and Italy through a complex process known as ‘exploitation competition’ [17]. In the UK, this process is enhanced by the squirrelpox virus (SQPV) [18,19]. The SQPV, belonging to the Poxviridae family, causes severe and often fatal disease in red squirrels [20]. By contrast, infected grey squirrels generally act as healthy carriers without exhibiting clinical signs of the disease [21,22]. Therefore, the replacement of the native species by SQPV-positive grey squirrels occurs faster than with exploitation competition alone [19,23]. The SQPV has spread into several countries, such as Canada [24], England, Ireland [25], Wales, and Scotland [26], but there is no evidence of it in grey squirrels introduced in Italy [27]. Squirrels can host many other pathogens, such as dermatophytes [28,29], yeasts [30], *Chlamydia* sp. [31], *Toxoplasma gondii* [32,33,34], *Leptospira* sp. [35,36], *Francisella tularensis* [37], *Borrelia* sp., *Babesia* sp., and *Rickettsia* sp. [38,39,40], and infections with adenoviruses are documented in Scotland [41], Germany [42], Northern Ireland [43], and Italy [44].

The expansion of the grey squirrel population was recognised as a threat to the Eurasian red squirrel by the European Strategy on IAS [45], and it was included among the ‘100 World’s Worst Invasive Alien Species’, a list compiled by the Global Invasive Species Database and maintained by the Invasive Species Specialist Group of the International Union of the Conservation of Nature. In particular, the European Union (EU) Regulation 1143/2014 aimed to prevent and manage the introduction and spread of IAS to protect biodiversity and ecosystem services, with close attention to the alien species included in a list of Union concern, called ‘the Union list’. The EU Regulation introduced a general ban on trade, possession, transport, and introduction of these species into the wild and imposed an obligation to immediately report, control, or eradicate when found. It also encouraged EU countries to activate a system of surveillance and monitoring of IAS of Union list concern and to identify the main pathways of introduction of invasive species, adopting at least one action plan to prevent the risk of further introductions. The grey squirrel was included in the Union list because of its already established invasiveness.

The LIFE13 BIO/IT/000204 Project: ‘Management of grey squirrel in Umbria: conservation of red squirrel and preventing loss of biodiversity in Apennines’ [46], was set up for the conservation of the Eurasian red squirrel in Umbria [47], using several steps: monitoring and eradication of invasive alien species, monitoring of native species, and raising public awareness through environmental education. The C4 action of this project provided information on the health status of *Sciurus carolinensis*, suggesting its relationship with other wildlife species and its role as a carrier of zoonotic agents. The presence of non-native squirrels in popular areas and their greater confident behaviour with humans could have critical implications on public health. Dermatophytoses are one of the most frequent skin diseases of humans and animals. A closely relation between them, especially in urban parks, could favour the onset of the disease by direct or indirect contact. This happens particularly in children and vulnerable subjects of all ages with impaired immune function. To achieve the right form of awareness, it is important to educate communities about zoonoses [48,49,50,51] to prevent further zoonotic introductions. Health status evaluation also provided helpful feedback for the management plan (i.e., identify areas where removal is a priority [47]).

## 2. Materials and Methods

### 2.1. Trapping Strategies, Study Area, and Sampling for Health Evaluation

A total of 1070 grey squirrels were removed since October 2014 to September 2018 from the study area of the LIFE U-SAVEREDS project, as described below. Mechanical Tomahawk live traps (mod. 202.5 Tomahawk Live Trap Co., Hazelhurst, WI, USA) baited with walnuts were placed opportunistically or following a regular network, according to the characteristics of each trapping site. They were numbered, geo-referenced, tagged, and placed at the base of tree trunks or on tree branches to avoid disturbance and entry of other wild or domestic species as much as possible. Moreover, they were placed away from sunlight or busy roads to limit additional stress to the captured squirrels and were checked at least twice a day. 

**Figure 1:**
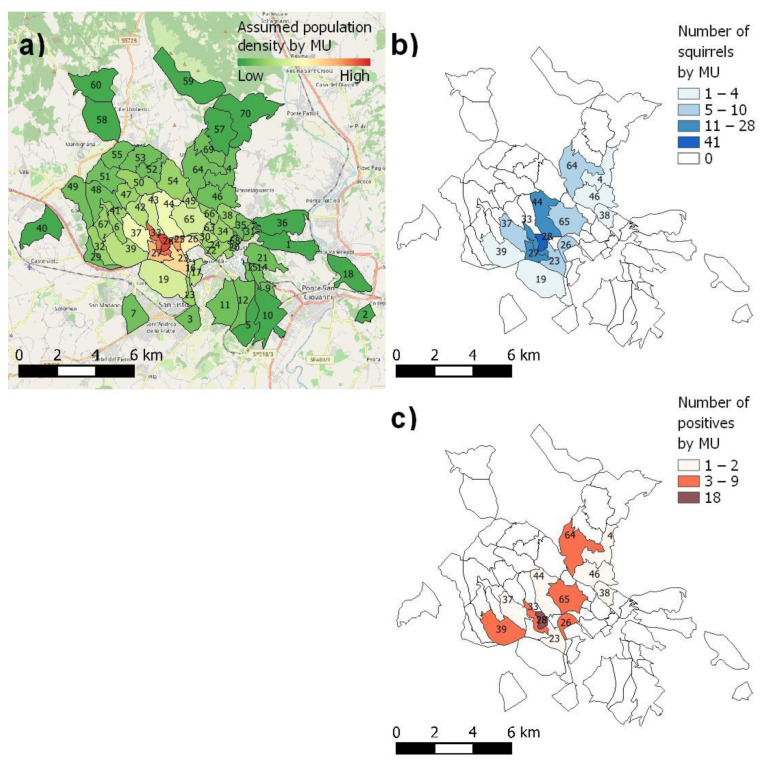
(**a**) Management units (MUs) and assumed grey squirrel population density moving away from the site of release, (**b**) number of squirrels by MU really submitted to health status evaluation, and (**c**) number of dermatophyte-positives squirrels by MU.

A minimum of 271 squirrels was established to be submitted to the health evaluation. The total number was calculated using *WIN EPISCOPE*, version 2.0 software [52], estimating a grey squirrel population of approximately 1500 members, with a 95% confidence level and 1% hypothesised pathogen prevalence. They were divided into a total of 70 management units (MUs), assuming a decreasing population density when moving away from the site of release (Figure 1a). The MUs were defined considering anthropogenic and habitat features and the known grey squirrel distribution, and they represented the study area of the LIFE U-SAVEREDS project. 

The management plan was carried out in accordance with the 92/43/CEE Directive, European Union Regulation No. 1143/2014, and the National Hunting Law 157/92 (Italy), with positive evaluation by the Italian Institute for Environmental Protection and Research, ISPRA (Istituto Superiore per la Protezione e la Ricerca Ambientale).

Euthanasia was performed using carbon dioxide, following the 1099/09/EC Regulation and American Veterinary Medical Association guidelines on animal welfare [53]. This method is classified as ‘hypoxia attributable to depression of vital centres’, such as the cerebral cortex, subcortical structures, and myocardium. Blood samples were collected immediately for subsequent serological investigations. Additionally, body weight, sex, and reproductive morphological conditions, including testicle and scrotum position [54] and vulva health [15], were recorded for each euthanised squirrel to track the potential growth of the *Sciurus carolinensis* population. Each carcass was then placed in a sealed plastic bag and subjected to necropsy at the Istituto Zooprofilattico Sperimentale dell’Umbria e delle Marche ‘Togo Rosati’ (IZSUM). Table 1 lists the pathogens investigated and the diagnostic techniques used for the health evaluation, including bacteriological, mycological, molecular, and serological analyses. The traps and handling materials were washed with Rely+On^TM^ Virkon^TM^ (LANXESSTM, Sudbury, England) and sterilised under UV light.

### 2.2. Bacteriological and Virological Examinations

Rectal swabs and pulmonary, central nervous system (CNS), and hepatic samples were aseptically collected for standard bacteriological examinations (Table 1). The samples were placed on MacConkey, mannitol salt agar, and sheep blood agar plates for aerobic incubation at 37 °C for 3 days. Suspicious colonies were isolated and then identified by biochemical reactions (API^®^ ID, bioMérieux, Craponne, France). For anaerobic incubation, samples were also spread on sheep blood agar plates placed in an anaerobic jar with AnaeroGen Compact (Oxoid Limited^®^, Basingstoke, England) and incubated at 37 °C for 18–24 h. Incubation was prolonged for one more day if no colonies appeared within 24 h. 

Viral isolation was carried out from oral swabs on Vero cell cultures (ATCC CRL-1586 VERO C1008) at the Biosafety Level 3 (BSL-3) laboratory at the Istituto Zooprofilattico Sperimentale Abruzzo e Molise ‘Giuseppe Caporale’ (IZSAM). Vero cells were grown in Dulbecco’s modified Eagle’s medium (DMEM) supplemented with antibiotics and 10% foetal calf serum. Monolayer cultures were inoculated with swab material and examined daily for the evidence of cytopathic effect (CPE) for 5–6 days. Three subsequent blind passages were carried out for each sample to allow CPE to appear. 

### 2.3. Mycological Examinations

Mycological examinations were performed to investigate the dermatophytes and yeasts (Table 1). Hair samples were collected using the Mackenzie technique [55], inoculated onto Dermasel agar, incubated at 25 ± 1 °C, and observed daily. Dermatophyte isolation was based on macroscopic and microscopic features [56], and reliable species identification was achieved through molecular-based techniques. Colonies grown on Dermasel agar were subjected to DNA extraction using the QIAamp DNA mini kit (QIAGEN^®^, Hilden, Germany) following a modified Gram-positive protocol (Appendix D: Protocols for Bacteria, Isolation of genomic DNA from Gram-positive bacteria), and subjected to a hemi-nested PCR test [57,58]. The PCR products were analysed by gel electrophoresis on a 2% agarose gel and observed using Midori Green Advance DNA stain (NIPPON Genetics Europe GmbH^®^). PCR-positive products were purified using the QIAquick PCR Purification Kit (QIAGEN^®^). Sequencing was performed using the BigDye Terminator v1.1 Cycle Sequencing Kit (Thermo Fisher Scientific^®^, Waltham, MA, USA), and reactions were separated through an ABI PRISM 310 Genetic Analyzer (Applied Biosystems^®^, Waltham, MA, USA). Consensus sequences were created by *BioEdit Sequence Alignment Editor*, version 7.0.9.0 software and aligned against those from the GenBank database.

Oral, rectal, and vaginal swabs were collected from each animal and plated on Sabouraud dextrose agar, followed by incubation at 37 °C for 24 h. Fungal growth attributable to yeasts was further investigated using API^®^ ID 32 C (Biomérieux^®^, Marcy-l’Étoile, France). 

### 2.4. Molecular Analysis

Genomic DNA and viral RNA were extracted from the spleen and liver samples using the High pure PCR template preparation kit (Roche Diagnostics GmbH^®^, Penzberg, Germany) and the QIAamp Viral RNA Mini Kit (QIAGEN^®^), respectively. As listed in Table 1, end-point PCRs were carried out to detect *Rickettsia* sp. [59], *Babesia* sp. [60], *Anaplasma* sp. [61], and adenovirus [62], whereas qPCRs were performed to investigate *Coxiella burnetii* [63] and *Borrelia burgdoferi sensu latu* [64]. Samples which tested positive for *B. burgdoferi sensu latu* were confirmed through end-point PCR tests [65] and then subjected to Sanger sequencing for species identification. DNA templates, obtained from squirrel eyelids using the QIAGEN DNeasy Mini Kit (QIAGEN^®^), were subjected to Pox Virus-specific nested PCR tests [66]. Moreover, nested RT-PCR and qRT-PCR tests were carried out for the *Hepatitis E* Virus (HEV) [67] and *West Nile* Virus (WNV) [68] detection, respectively.

### 2.5. Serological Analysis

Antibodies specific for *Toxoplasma gondii*, *Leptospira interrogans*, *Francisella tularensis*, *Chlamydia* sp., hantaviruses, and flaviviruses were investigated in the serum of each captured animal (Table 1). 

**Table 1:** Details for bacteriological, mycological, molecular, and serological investigations.

	Pathogen	Matrix	Diagnostic Technique	Additional Investigations	PCR References
Bacteriological investigations	Bacteria	Rectal swab Lung Brain Liver	Standard Bacteriological examination	/	
Mycological investigations	Dermatophytes	Fur (hair)	Mycological exam	Hemi-nested PCR from fungal colony + sequencing	[57,58]
	Yeasts	Oral swab Rectal swab Vaginal swab	Cultural exam	/	
Molecular investigations	*Rickettsia* spp.	Spleen Ticks	End-point PCR	/	[59]
	*Babesia* spp.	Spleen Ticks	End-point PCR	/	[60]
	*Anaplasma* spp.	Spleen Ticks	End-point PCR	/	[61]
	Adenovirus	Liver Intestine	End-point PCR	/	[62]
	*Coxiella burnetii*	Spleen Ticks	qPCR	/	[63]
	*Borrelia* *burgdoferi s.l.*	Spleen Ticks	qPCR	End-point PCR + sequencing	[64,65]
	Poxvirus	Eyelid	Nested PCR	/	[66]
	*Hepatitis E* Virus (HEV)	Spleen Ticks	Nested RT-PCR	/	[67]
	*West Nile* Virus (WNV)	Spleen Ticks	qRT-PCR	/	[68]
Serological investigations	*Toxoplasma gondii*	Serum	Latex test	/	
	*Leptospira* *interrogans*	Serum	MAT	/	
	*Francisella tularensis*	Serum	MAT	/	
	*Chlamydia* spp.	Serum	CFT	/	

A latex agglutination test was used to detect anti-*T. gondii* antibodies (Toxo Latex Kit, Rapid Labs Ltd.^®^, Colchester, England [69]) by mixing an equal volume of each serum sample with toxoplasma-sensitised latex particles on a glass slide. Agglutination was determined visually under high-intensity light, and samples which caused any degree of agglutination of the toxoplasma-sensitised latex were considered positive.

Serological diagnosis of *L. interrogans* was performed using the microagglutination test (MAT). Each serum sample was tested against eight locally circulating *L. interrogans* serovars, namely, *icterohaemorrhagiae*, *canicola*, *tarassovi*, *bratislava*, *grippotiphosa*, *ballum*, *hardjo*, and *pomona*. Serum was considered positive at a 1:100 dilution for the tested antigen if at least 50% of Leptospira were agglutinated compared with a control antigen. MAT was also used for the serological diagnosis of *F. tularensis*, considering positive titres of ≥1:128 [10].

Anti-*Chlamydia* sp. antibodies were investigated by using the complement-fixation test with standardised Serion CFT reagents, according to the manufacturer’s instructions. Sera with 50–100% inhibition of haemolysis at a 1:10 dilution were considered positive.

Serological tests for hantaviruses, particularly the *Puumalavirus* (PUUV) and *Dobravavirus* (DOBV), were performed using the ReaScan Ab-Dect PUUMALA IgG immunochromatography test and the Ab-Dect Dobrava-Hantaan IgG EIA ELISA kit (both Oy Reagena Ltd.^®^, Toivala, Finland), respectively, following the manufacturer’s instructions. Serological assays for flaviviruses were carried out using the competitive ELISA INgezim WNV Compac (Ingenasa^®^, Madrid, Spain), which cross-reacts with different flaviviruses, and the positive samples were further analysed by using serum neutralisation (SN) assays for WNV and Usutu virus (USUV). 

### 2.6. Statistical Analysis

The Fisher’s exact test was used to assess differences in positivity by season, gender, and presence of lesions. The Pearson’s Chi-square test was used to evaluate the differences in positivity for MUs. Statistical significance was set at *p* < 0.05. All analyses were performed using the Stata software (StataCorp. 2019. Stata Statistical Software: Release 16. College Station, TX, StataCorp LLC).

## 3. Results

Out of the 271 foreseen, a total of 154 squirrels (82 males and 72 females) were subjected to necropsy and health investigations. The discrepancy between the hypothesised and the analysed squirrels was because they were not distributed over all 70 MUs, as initially considered. Most trapped animals appeared to be healthy and free from macroscopic internal or external lesions, except eight animals (5.2%) that showed non-exudative alopecic lesions.

Dermatophytes were found in 48 squirrels (31.2%), comprising 30 males and 18 females (Table 2). Conventional methods supported by molecular-based techniques allowed the identification of four different species: *Nannizzia racemosa* (*n* = 34, 70.8%), *Trichophyton mentagrophytes* (*n* = 7, 14.6%), *Trichophyton thuringiense* (*n* = 4, 8.3%), and *Trichophyton ajelloi* (*n* = 3, 6.3%). The ITS sequence analysis showed a degree of similarity between 99% and 100%. No significant differences were found between being tested positive for dermatophytes and the presence of lesions (*p* = 0.257) or gender (*p* = 0.163). Dermatophyte-positive squirrels were more likely to be found in autumn (*p* = 0.027) than in other seasons (spring: *p* = 0.273, summer: *p* = 0.058, winter: *p* = 0.384). Moreover, the presence of dermatophyte-positive squirrels was found to be higher in MU 28 than in the other MUs (*p* = 0.04, Figure 1c).

*Borrelialusitaniae*, the causative agent of Lyme’s disease, was detected in a *N. racemosa*-positive squirrel, originating from MU 26. *Coxiella burnetii*, the Q fever causative agent, was detected in two *N. racemosa*-positive squirrels, originating from MU 28. None of the remaining infectious agents were found on molecular analysis.

*Candida albicans* was detected in oral, rectal, and vaginal swabs collected from four animals originating from different MUs (27, 38, 46, 64), which tested negative for dermatophytes. Moreover, *Escherichia coli* was isolated from 180 rectal swabs, but none showed virulence factors. No other yeasts or bacteria were isolated through mycological or bacteriological analysis. CPE was not observed in any of the oral swabs. Serological analyses did not detect antibodies specific to any of the investigated pathogens except for flaviviruses. Five serum samples tested positive in the ELISA test for WNV, but all tested negative in the confirmatory SN assays for WNV and USUV. 

The results suggested a potential role as a zoonotic carrier for the Eastern grey squirrel, supporting the importance of its management to guarantee human health. Moreover, information obtained by this management plan reinforces the surveillance of pathogens for humans but also for domestic animals and livestock (e.g., West Nile virus, *Babesia* sp., *Borrelia* sp., *Leptospira* sp., etc.) as well as for wild native fauna (e.g., *Adenovirus*, *Poxvirus*, *Chlamydia* sp., etc.). 

## 4. Discussion

LIFE13 BIO/IT/000204–LIFE U-SAVEREDS was established for Eurasian red squirrel conservation in Umbria through the eradication of invasive alien species [47]. The C4 action of the project provided information on the health status of *S. carolinensis*. Health status evaluation represents an important tool for identifying infectious agents and providing helpful feedback for the management and eradication procedures. 

Despite their disease-free appearance, the Eastern grey squirrel was identified as a potential source of zoonoses due to a high percentage of dermatophyte-infected individuals (31.2%) found in 12 MUs (Table 2). Molecular analysis ensured reliable identification of the fungal species, as some strains were indistinguishable by morphological and microscopic features. The isolated dermatophytes are zoophilic (*T. mentagrophytes*) and geophilic (*N. racemosa*, *T. ajelloi*, and *T. thuringense*) [70]. According to other authors, this may pose a health risk for humans through direct contact with these animals or environmental contamination [56]. *T. mentagrophytes* var. *interdigitale* (previously identified as *T. mentagrophytes*) has been previously reported in squirrels [56,71]. Dermatophytes have also been recovered in other wild species belonging to different geographic areas and taxa, such as rodents, insectivores, lagomorphs, wild boars, foxes, wolves, polecats, badgers, and chamois, which have been observed both as healthy carriers and, more rarely, as affected by clinical ringworm [72,73,74,75,76]. In this study, among the eight squirrels which tested positive for dermatophytes, only four of them showed alopecic lesions (Table 2) that could be caused by traumatic events, such as being captured in mechanical iron traps. Among the statistical parameters considered, the seasonal count was the only one of significance: positive squirrels were more likely to be found in autumn (*p* = 0.027) than in the other seasons, probably because of favourable climatic conditions, such as wet weather and more food availability. Moreover, the greatest number of dermatophyte-positive squirrels found in MU 28 could be presumably due to the overpopulation in this area, which represented the site of the release of the Eastern grey squirrel in Umbria. 

*Borrelia lusitaniae* and *Coxiella burnetii* detection also confirmed that the Eastern grey squirrel could represent a potential source of zoonoses, because as tick-borne disease agents, they carry ticks that occasionally bite humans. Tick samples previously collected from wild rodents in central Italy were positive for *B. burgdoferi sensu latu* and *C. burnetii* [77]. The squirrels examined were not infested by ticks, but it is also probable that ticks dropped off the animals prior to capture. In light of this finding, *B. lusitaniae* positivity in squirrels could be related to dogs which came to the public park, especially in MU 26, as it is known that several species of ticks, such as *Ixodes* sp., are hosted by companion animals and wild animals, such as flying squirrels [78]. However, *C. burnetii* detection could be attributed to contact with infected faeces and birth products [77]. 

The Veterinary Police Regulation (Presidential Decree 320/54) and the information system of infectious and diffusive diseases (Ministerial Decree 15 December 1990) differentiate notifiable from non-notifiable diseases, providing useful management feedback. Lyme’s disease is not considered a notifiable disease—the management response was limited to the MU where the disease occurred, increasing the sample size for sanitary investigations. In contrast, dermatophytes and Q fever are defined as notifiable diseases; in these cases, both an individual-dependent response and an area-dependent response were applied. An individual-dependent response indicated an increase in the catch rate and restrictions for individual squirrel management. Activities such as the translocation of sterilised grey squirrels or the release of Eurasian red squirrels were also foreseen within the project. However, based on the results of the sanitary screening, no sterilised squirrels could be moved to/from the MUs where those pathogens existed, and neither could Eurasian red squirrels be released there (area-dependent response).

No evidence of hantavirus was reported in the study area, whereas positive results from the serological test for flavivirus may indicate circulation of any flavivirus other than WNV and USUV among squirrels. 

Since all the swabs (oral, rectal, and vaginal) collected from four animals tested positive for *C. albicans,* their immune systems were probably compromised, but the low prevalence was not considered alarming for public health. This included the incidence of *E. coli,* which was not septicaemic or haemolytic, and was thus treated as commensal intestinal bacteria. 

In particular, the authors noticed that all the analysed squirrels were found to be negative for the presence of SQPV DNA, establishing no evidence of its infection in Italy [27] and preventing a more rapid replacement of the native species [19]. 

## 5. Conclusions

This survey on the Eastern grey squirrel population indicated that it is necessary to raise awareness of its impacts on biodiversity and human and animals’ health. The presence of this IAS in the Umbria region causes ecological damages through different processes, including competition, predation, parasitism, disease vectoring, hybridisation, and spillover/spillback mechanisms. In particular, the ‘exploitation competition’ mentioned previously is a complex process that occurs as a result of the concurrent presence of *S. carolinensis* and *S. vulgaris*. Naturally, the squirrel native species lives in balance with the local flora (e.g., hazelnut and oak groves), whereas the non-native species is responsible for injuries in nuts’ harvesting and debarking of trees, resulting in severe economic losses. Moreover, due to their size, Eastern grey squirrels can prey upon nestlings, increasing biodiversity damage. In addition, their confident behaviour can be dangerous for humans, because they can be reservoirs of zoonoses such as dermatophytoses and tick-borne diseases. The LIFE U-SAVEREDS strategy could be suitable to achieve the right form of awareness about the previously discussed topics, in particular through environmental educational plans and scientific dissemination that involve the community. 

Thanks to the eradication campaign, *S. vulgaris* is currently less life-threatened by *S. carolinensis*: the Eurasian red squirrel seems to have almost completely recovered its natural distribution area, as proven by the post-LIFE monitoring (data not yet published). 

Other wild species could be investigated by similar methods to obtain interesting epidemiological data and adopt management advice to guarantee both ecological balance and human and animals’ health. 

## Figures and Tables

**Table 2:** Results and details of detected dermatophytes, related to the presence of other pathogens.

MU	Analysed Samples	Positive Samples	SEX	Accession Number	Skin Lesions	Other Pathogens
**4**	4	1	1 M	*N. racemosa* (EF631613)	No	
**19**	1	0		/	No	
**23**	7	1	1 F	*T. thuringiense* (KT428771)	Yes	
**26**	10	3	2 M 1 F	*N. racemosa* (EF631613) *N. racemosa* (EF631613)	No No	*B. lusitaniae*
**27**	15	0		/	No	
**28**	41	18	2 M 1 M 5 M 5 F 2 M 1 F 2 M	*N. racemosa* (EF631613) *N. racemosa* (EF631613) *N. racemosa* (EF631613) *N. racemosa* (EF631613) *T. mentagrophytes* (AB566303) *T. mentagrophytes* (AB566303) *T. ajelloi* (ATCC 28454)	No Yes No No No No No	*C. burnetii*
**33**	28	9	3 M 3 F 1 M 1 M 1 F	*N. racemosa* (EF631613) *N. racemosa* (EF631613) *T. mentagrophytes* (AB566303) *T. mentagrophytes* (AB566303) *T. mentagrophytes* (AB566303)	No No Yes No No	
**37**	8	2	1 M 1 F	*N. racemosa* (EF631613) *T. thuringiense* (KT428771)	No No	
**38**	3	2	2 M	*N. racemosa* (EF631613)	No	
**39**	4	3	3 F	*N. racemosa* (EF631613)	No	
**44**	16	1	1 M	*T. mentagrophytes* (AB566303)	Yes	
**46**	3	2	1 M 1 F	*T. thuringiense* (KT428771) *N. racemosa* (EF631613)	No No	
**64**	8	3	2 M 1 F	*N. racemosa* (EF631613) *T. thuringiense* (KT428771)	No No	
**65**	6	3	2 M 1 M	*N. racemosa* (EF631613) *T. ajelloi* (EF631614)	No No	
	**154**	**48** (31.2%)				

## Data Availability

Data are available upon request.
